# Best-Quality Vessel Identification Using Vessel Quality Measure in Multiple-Phase Coronary CT Angiography 

**DOI:** 10.1155/2016/1835297

**Published:** 2016-09-19

**Authors:** Lubomir Hadjiiski, Jordan Liu, Heang-Ping Chan, Chuan Zhou, Jun Wei, Aamer Chughtai, Jean Kuriakose, Prachi Agarwal, Ella Kazerooni

**Affiliations:** Department of Radiology, University of Michigan, Ann Arbor, MI 48109, USA

## Abstract

The detection of stenotic plaques strongly depends on the quality of the coronary arterial tree imaged with coronary CT angiography (cCTA). However, it is time consuming for the radiologist to select the best-quality vessels from the multiple-phase cCTA for interpretation in clinical practice. We are developing an automated method for selection of the best-quality vessels from coronary arterial trees in multiple-phase cCTA to facilitate radiologist's reading or computerized analysis. Our automated method consists of vessel segmentation, vessel registration, corresponding vessel branch matching, vessel quality measure (VQM) estimation, and automatic selection of best branches based on VQM. For every branch, the VQM was calculated as the average radial gradient. An observer preference study was conducted to visually compare the quality of the selected vessels. 167 corresponding branch pairs were evaluated by two radiologists. The agreement between the first radiologist and the automated selection was 76% with kappa of 0.49. The agreement between the second radiologist and the automated selection was also 76% with kappa of 0.45. The agreement between the two radiologists was 81% with kappa of 0.57. The observer preference study demonstrated the feasibility of the proposed automated method for the selection of the best-quality vessels from multiple cCTA phases.

## 1. Introduction

Coronary CT angiography (cCTA) is a useful noninvasive modality for imaging of the heart and evaluation of the extent of plaques. However, due to the coordinated motion of the heart chambers, different arterial segments may be blurred at different phases of the cardiac cycle [1]. To reduce this artifact, electrocardiographic (ECG) gating is employed for the acquisition of cCTA and the cCTA examinations are reconstructed at multiple cardiac phases. In this way, each of the coronary arterial segments has a better chance to be captured in a stationary and good-quality state in at least one of the phases. However, the search for atherosclerotic plaques in multiple-phase cCTA volumes is time consuming for radiologists or a computer-aided detection (CAD) system.

A number of studies reported methods of automatic selection of the best-quality phase of the entire coronary arterial tree or motion correction during reconstruction of cCTA or 3D angiograms. Rasche et al. [2] proposed an algorithm for automatic selection of the optimal cardiac phase for image reconstruction in vascular interventional X-ray imaging using a C-arm system. The algorithm was based on the analysis of a four-dimensional data set using an image quality index. The performance of the algorithm was evaluated in eight porcine models. Hoffmann et al. [3] evaluated an algorithm for automatic phase selection in cCTA based on the concept of motion maps. The motion-map phase selection approach was compared to that with manual iterative selection in cCTA of 20 patients. A high level of agreement was found between the two approaches with better results in patients with low heart rates. Joemai et al. [4] compared fixed, manual, and automatic phase selection methods in cCTA. The automatic method estimated a motion map from the raw data and reconstructed the phase with the least motion. The difference in the selections between the manual and the automatic methods was not statistically significant. In a different study, Joemai et al. [5] also compared the manual and the automatic method for optimal phase selection in cCTA for the assessment of the global left ventricular function. They concluded that the automated phase selection method was similar to the manual selection. Ruzsics et al. [6] compared a commercial system for automatic selection of the cardiac phase with the least motion for cCTA reconstruction with manual selection. No statistically significant difference was found between automatic and manual selections. Rohkohl et al. [7] also developed a method in which two motion artifact metrics were used to guide the automatic adjustment of the motion field parameters for motion-compensated reconstruction to minimize motion artifacts. The image quality improvement of the MAM optimization was visually confirmed in four cCTA patient cases.

The above studies focused on selection of the best-quality cCTA phase, which inevitably have to make compromise among the individual arteries because there may not be a single phase in which all arteries attain their best quality. Lessick et al. [9] proposed and evaluated a vessel-specific method which automatically output the minimum motion phase for each of the three main coronary arteries. The study demonstrated that multiple phases were required to ensure optimal image quality for all three coronary arteries and that a vessel-specific phase selection algorithm achieved superior results to the standard global approach. We are developing an alternative novel approach, in which an automated method will select the best-quality phase for any individual arterial segment on the coronary arterial trees from the available phases of a cCTA study during radiologists' interpretation or computerized analysis. The selected arterial segments may be considered to be components of a virtual single best-quality coronary arterial tree. The selection process will be carried out in the background, transparent to the radiologist. When the radiologist reads each individual arterial segment, the best-quality phase of that segment will be automatically displayed in any preferred format(s) (straightened or curved multiplanar reformation, original axial slices) on a clinical workstation for the radiologist's interpretation. We have previously developed the vessel segmentation and registration methods [10–12]. We have reported initial pilot results based on the idea of automatic vessel selection in a conference [13]. In the current paper, we focus on the implementation framework and the methodologies for the identification of corresponding vessels from multiple phases and the selection of the best-quality vessel in much greater detail using a larger data set. We also demonstrated the feasibility of the automated best-quality vessel selection method by an observer preference study and statistical analysis.

## 2. Materials and Methods

**Figure 1:**
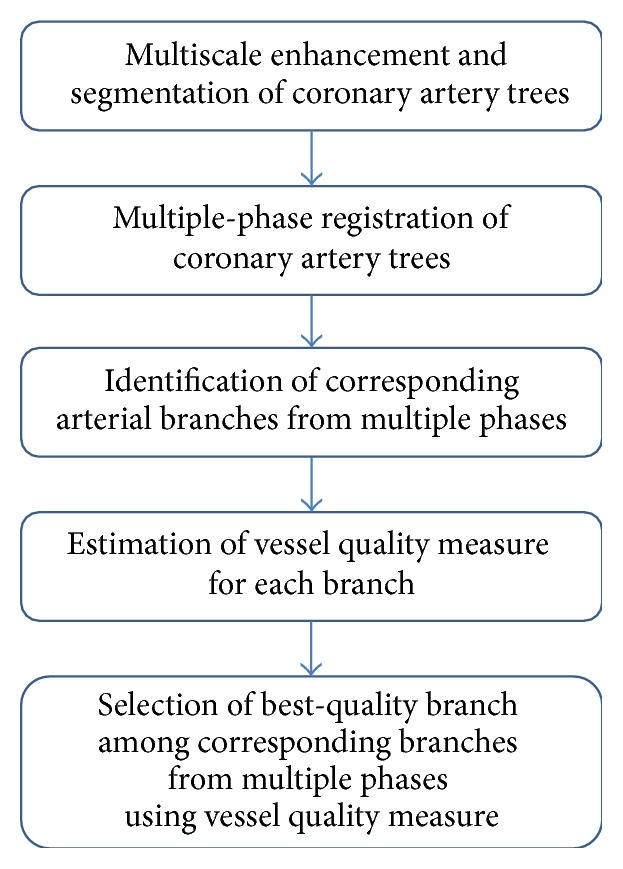
Multistage framework for selection of the best-quality phase for individual arterial branches in multiphase cCTA.

The multistage framework for automated best-quality vessel selection in cCTA is shown in Figure 1. The left and right coronary artery trees (LCA and RCA) are segmented and tracked from each phase of the reconstructed cCTA volume [10, 11]. The LCA and RCA trees are separately registered to their respective trees in the multiple phases [12]. The corresponding arterial branches from the registered phases are identified. A vessel quality measure (VQM) is calculated from each branch and the branch with the highest VQM among the multiple phases is considered to be the best-quality phase of this arterial branch. The steps of the process are described in the following sections.

### 2.1. Coronary Arterial Tree Segmentation and Registration

**Figure 2:**
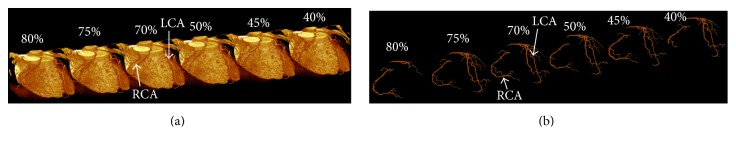
cCTA scans acquired with ECG-gating and reconstructed at multiple cardiac phases: (a) 3D rendered cCTA volumes for 6 acquired cardiac phases (80%, 75%, 70%, 50%, 45%, and 40%). Left (LCA) and right (RCA) coronary arterial tree in phase 50% are marked by white arrows. (b) Segmented LCA and RCA tree in each phase (80%, 75%, 70%, 50%, 45%, and 40%) using multiscale enhancement and dynamic balloon tracking method [10, 11].

**Figure 3:**
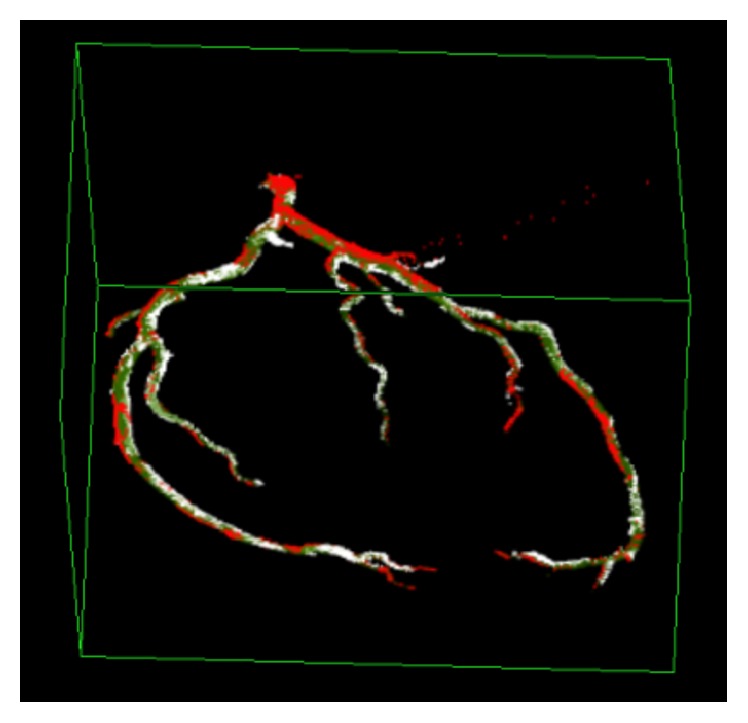
LCA trees from Figure 2 which are automatically registered by using nonlinear CBSO and AQSO methods [12]. The coronary trees in phases 70% and 80% were registered first (70%-80%). Then by using the CBSO method the coronary tree in phase 75% was registered to 70%-80% to obtain the 70%-80%-75% tree. By following the same approach the coronary trees in phases 45% and 50% were also registered (45%-50%). Again, by using the CBSO method, the coronary tree in phase 40% was registered to 45%-50% to obtain the 45%-50%-40% tree. The 45%-50%-40% tree was then registered to the 70%-80%-75% tree by using the AQSO method followed by the CBSO method. In the image, the reference (unwrapped) coronary tree is shown in red, the warped coronary tree in white, and the overlap between the trees in green.

A cCTA examination acquired with ECG-gating and reconstructed at 6 cardiac phases (e.g., 80%, 75%, 70%, 50%, 45%, and 40%) is shown in Figure 2(a). For each phase, a 3D rendered volume of interest enclosing the heart region in the cCTA is presented. The coronary arteries are segmented in each phase, as shown in Figure 2(b) by our multiscale coronary artery response and rolling balloon region growing and tracking method [10, 11]. The centerlines of the segmented arterial trees are also determined during rolling balloon vessel tracking. The arterial trees from the different phases are then coregistered by a multistep registration method developed in our laboratory, which uses cubic B-spline with fast localized optimization (CBSO) and an affine transform with quadratic terms and nonlinear simplex optimization (AQSO) [12]. A coregistered left coronary artery tree combining six phases is shown in Figure 3. Details of the coronary artery tree segmentation and multiple-phase registration methods and their performances can be found in our previous publications [10–12].

### 2.2. Identification of Coronary Tree Branches and Determination of Correspondence

**Figure 4:**
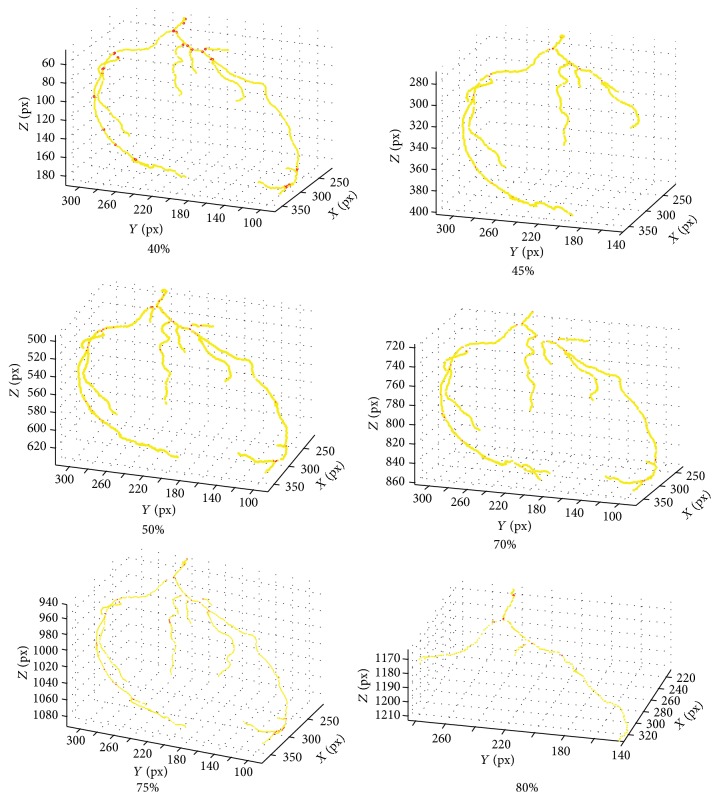
Automatically detected vessel branching points (in red), which are propagated to all phases.

To identify the coronary tree branches, the first stage is the determination of the corresponding branching points. The branching points of the coronary trees in every phase are automatically detected (Figure 4) as a part of the balloon tracking method. Every detected branching point in a given phase is then projected to the registered coronary trees of the other phases. This procedure is implemented in order to (1) increase the likelihood of consistent identification of the branching points in every phase because the detection of branching points on a coronary tree in a low-quality phase is challenging and branching points may be missed and (2) establish the correspondence between the branching points from different phases.

**Figure 5:**
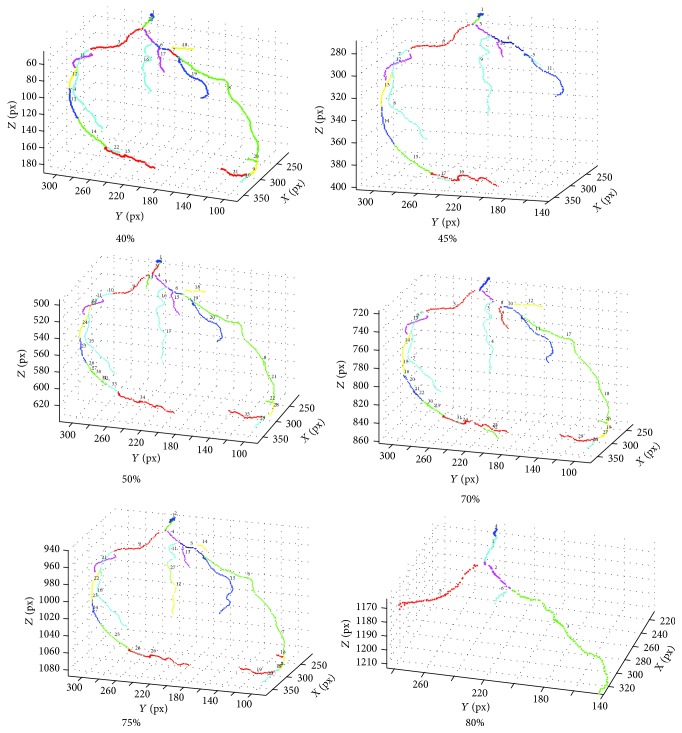
LCA tree: automatically identified corresponding vessel branches (shown by matching colors) in six phases. Note that some colors are repeatedly used for different branches because of the limited number of colors available. The different branches of the same color can be distinguished by locations.

For a branching point in a given phase, the above process will generate multiple potential branching point candidates in its proximity, due to inaccuracies in the detection of branching point locations in the different phases, as well as the inaccuracies in the coronary tree registration. Therefore, we developed a method to automatically identify the most likely branching point in a given phase and then identify the corresponding branching points in all phases (Figure 4) by using the combined coregistered tree and distance criteria. A projected branching point from a different phase will be kept only if the distance between the point and any existing branching point in the current phase is larger than *d*, where *d* was chosen to be 20 voxels experimentally. If the distance between the projected branching point and the closest branching point in the current phase is smaller or equal to *d*, then a correspondence will be established between the branching point in the current phase and the original branching point from the different phase that generates the projected point. This process is applied to all branching points in all phases. The branching points are used to mark separate vessel branches and to split the coronary tree in each phase. The centerlines are also split into centerline branches following the vessel branch identification (Figure 5).

In the next step, the corresponding branches of the same vessel appearing in different phases are identified. The correspondence between the branches is established by using the following criteria:

(1) For every centerline voxel *c*_*h*_ of a branch *b*_*G*_^*i*^ within the coronary tree *C*_*G*_ at phase *G*, (*c*_*h*_ ∈ *C*_*G*_), the shortest distance to the centerline of the coronary tree *C*_*U*_ in another phase *U* after registration is calculated as

(1) $$\begin{array}{c} D\left(\;c_{h},C_{U}\;\right)=\min\left\{\;d\left(\;c_{h},t\;\right):t\in C_{U}\;\right\}, \end{array}$$

where the function* d* is the Euclidean distance. Let *t*_*h*,min_ be the voxel on* C*_*U*_, at which the shortest distance *D*(*c*_*h*_, *C*_*U*_) is found in branch *b*_*U*_^*j*^ and *D*(*c*_*h*_, *C*_*U*_(*t*_*h*,min_)) ≤ *θ*, where *θ* was set to be 3 voxels. If the shortest distance voxels, *t*_*h*,min_, for at least 70% of the centerline voxels *c*_*h*_ within the branch *b*_*G*_^*i*^, belong to the same branch *b*_*U*_^*j*^ in phase *U* and vice versa, then there exists a correspondence between the branches *b*_*G*_^*i*^ and *b*_*U*_^*j*^.

(2) Short branches that are subsets of longer branches in the same phase are ignored.

**Figure 6:**
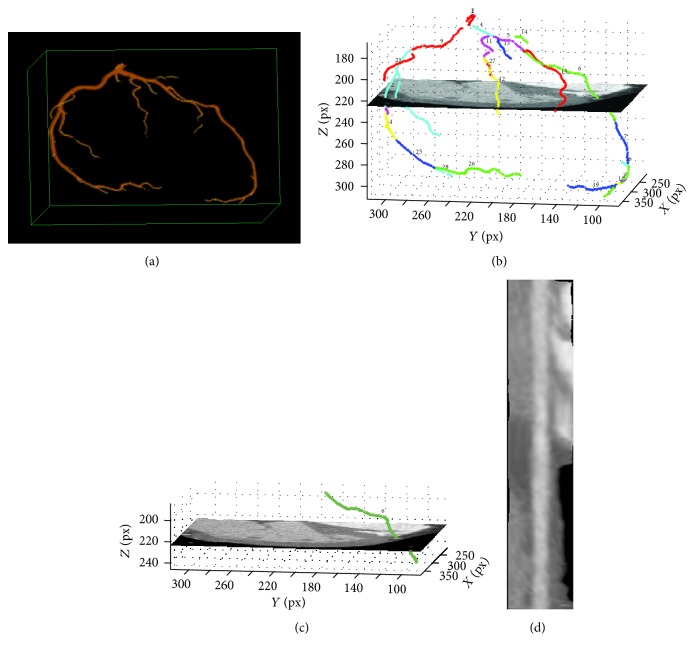
Vessel straightening. Curved planar reformation was used to straighten each of the branches.

The process is repeated for all phase pairs to determine the correspondence of all branches (Figure 5). This process is applied separately to the left and the right coronary trees. Each of the branches is then straightened by curved planar reformation (CPR), where every coronary vessel branch and a surrounding neighborhood in the cCTA volume is transformed to a rectangular volume by resampling the original cCTA volume in planar cross sections perpendicular to the branch centerline (Figure 6). Our implementation of the CPR method was described previously [14].

### 2.3. Vessel Quality Measure (VQM)

In this preliminary study, we defined a simple vessel quality measure (VQM) to automatically estimate the vessel quality. First, the gradients *G*_*i*_ along the radii *R*_*i*_  (*i* = 1,…, *N*_*c*_*h*__) of the vessel cross section perpendicular to the centerline at the centerline voxel *c*_*h*_ of a given branch *b*_*G*_^*k*^ are calculated [14]:

(2) $$\begin{array}{c} G_{i}=I_{\text{in}}-I_{\text{out}}, \end{array}$$

where *I*_in_ is the average CT value at half radius from the vessel center *c*_*h*_ to the vessel wall, *I*_out_ is the average CT value from the vessel wall to a distance of half radius outside the vessel, and *N*_*c*_*h*__ is the number of the points along the vessel wall of the vessel cross section at *c*_*h*_. The radial gradient at each voxel *c*_*h*_ along the centerline of a branch *b*_*G*_^*k*^ is calculated as the mean of the gradients *G*_*i*_ along all radii *R*_*i*_ of the vessel cross section:

(3) $$\begin{array}{c} {\overset{-}{G}}_{c_{h}}=\frac{\sum_{i=1}^{N_{c_{h}}}G_{i}}{N_{c_{h}}}, \end{array}$$

**Figure 7:**
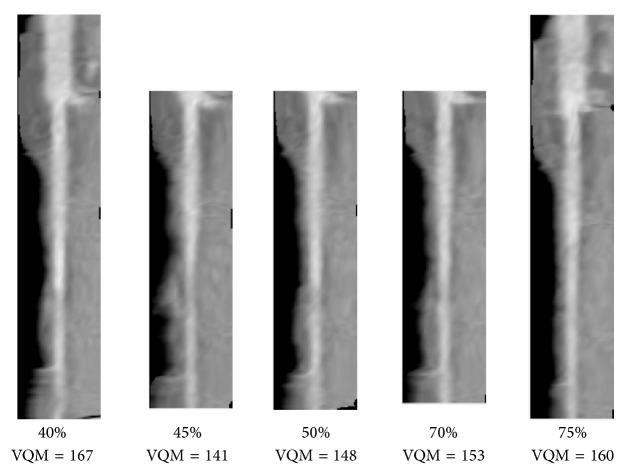
A straightened branch from the LCA tree in Figure 5 in five phases. The VQM was calculated for the corresponding straightened branches in all available phases. (The branch was missing in the 80% phase.)

Finally, a VQM is derived as the average of the radial gradient values over all centerline points of the vessel branch:

(4) $$\begin{array}{c} {\text{VQM}}_{b_{G}^{k}}=\frac{\sum_{h=1}^{M}{\overset{-}{G}}_{c_{h}}}{M}, \end{array}$$

where *M* is the number of voxels *c*_*h*_ along the centerline of a branch *b*_*G*_^*k*^. The VQM is calculated for every branch in every phase. In Figure 7, the VQM was calculated for the five corresponding CPR straightened branches in five of the phases. In one of the phases (80%), the coronary tree missed many of the arterial segments (Figure 4) and the branch in Figure 7 was one of the missing ones. The quality of the corresponding branches among all phases in a cCTA exam can then be ranked by their VQM values.

The distance between the point inside and the point outside the vessel in the denominator of the gradient calculation is not explicitly included in (2) because the VQM is designed to compare the relative vessel wall sharpness of the same vessel segment at multiple phases and the distance (i.e., the radius) is the same for all phases. Using the radius estimated at each angle or each center point of the vessel segment would introduce more variations due to noise so that a constant distance is assumed for the relative radial gradient estimation. We used the radial gradient averaged along the branch because it further reduces noise and is more robust than a point-by-point comparison.

### 2.4. Observer Study

**Figure 8:**
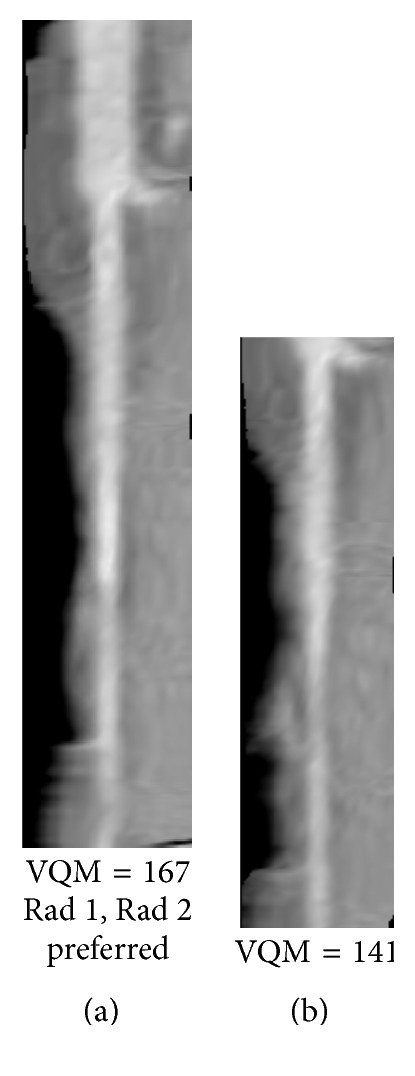
A pair of best- and worst-quality vessel branches selected automatically from the corresponding arterial branches in Figure 7 using VQM and the radiologists' preferences. The automated selection and the radiologists' preferences matched in this case (branch (a)).

We conducted an observer preference study to visually compare the relative quality of the vessels and compared with the automatic ranking with VQM. Because of the large number of possible vessel pairs that can be formed by exhaustive pairing of the corresponding vessel branches from multiple phases, to limit the radiologists' effort required for reading, we used a single pair of each vessel branch in our data set for the observer study. First, a pair of the best- and worst-quality branches among the available phases for each branch was automatically identified using the VQM (Figure 8). The pair of vessel branches was displayed side by side on two DICOM-calibrated high resolution monitors by a graphic user interface (GUI). The display of the best- and the worst-quality image as the left or the right image in a pair was randomized. An observer, blinded to the VQM, visually compared the pairs of corresponding branches and selected the image with better quality vessel based on his/her subjective judgment (Figure 8). The observer recorded his/her preference using an in-house developed GUI. Two experienced cardiothoracic radiologists with 10 and 7 years of experiences evaluating cCTA participated in the study as observers. Figure 8 shows an example for which the automated selection matched both radiologists' preference.

### 2.5. Data Set

With Institutional Review Board (IRB) approval, cCTA examinations for seven patients (2 men and 5 women; age range, 31–65 years; mean age, 49.4 years) were collected retrospectively from the patient files at the University of Michigan Health System and used in this preliminary study. The cCTA cases were acquired with a clinical protocol in which an isoosmolar nonionic contrast medium (Visipaque; GE Healthcare) was administered using an 18-gauge cannula in an upper extremity vein. A test bolus of 15 to 20 mL at the rate of 4 to 5 mL/s was administered with sequential scanning every 2 seconds at the level of the left main coronary artery, with a region of interest placed in the aortic root, to determine the optimum scan delay for each patient. For the coronary CT angiograms 80 mL of contrast medium was injected (60 mL at 5 mL/s and 20 mL at 3.5 mL/s) followed by a saline chase bolus of 50 mL at 5 mL/s. The mean and standard deviation heart rate of the patients were 62.7 ± 5.2 bpm.

The cCTA images were acquired by helical retrospective gating at 120 kVp and 440–800 mA with GE multidetector CT scanners (GE Healthcare Lightspeed VCT (6 patients) and Discovery CT750 HD (1 patient)). The cCTA image slices were reconstructed at 0.625 mm slice interval and 0.488 mm in-plane pixel size. The clinical protocol of cCTA in our department reconstructed 6 phases in a range from 40% to 87%. While more phases can be reconstructed from the data, 6 phases are used in the clinical protocol at our health system because they are clinically sufficient to cover a broad range of heart rates. This strategy provides optimal yield and a balance between diagnostic accuracy and efficiency. Routine reading of more than 6 phases is clinically impractical and overly burdens radiologists' workload. In our study, we tried to emulate clinical practice, where only select phases are generated for assessment but our method is applicable to more or fewer phases.

The data set therefore contained a total of 42 cCTA volumes (7 patients with 6 phase cCTA scans each) with 84 coronary arterial trees (42 LCA trees and 42 RCA trees). After automatic registration and identification, 167 groups of corresponding branches were established (102 LCA and 65 RCA). The VQM of 833 branches in 6 phases were calculated (531 LCA branches and 302 RCA branches). Note that not all vessel branches had a complete set of 6 phases because some vessels might be lost at segmentation and tracking due to poor image quality. For each group of corresponding branches, two branches, the branch with the highest VQM and the branch with the lowest VQM, were selected based on the VQM, as described in the previous section. This resulted in 167 branch image pairs (102 pairs from LCAs and 65 pairs from RCAs). Detailed information for the number of established branch image pairs for each case is given in Table 1.

### 2.6. Evaluation Methods

The performance of the automatic selection using VQM was evaluated by the following two methods:

1. Estimation of the percentage of the total number of vessel pairs for which the automatic selection agreed with the radiologist's selection of the higher quality branch in the pair.
2. Cohen's kappa statistics to estimate the agreement between the automatic selection and the radiologist's selection of the higher quality branch in the pair.

For comparison, the agreement between the two radiologists was also evaluated with the two methods.

## 3. Results

The overall agreement between radiologist 1 and the automated selection of the best-quality branches was 76% for the 7 cases (range: 53% to 93%). The overall agreement between radiologist 2 and the automated selection of the best-quality branches was 76% (range: 47% to 93%). The overall agreement between radiologist 1 and radiologist 2 was 81% (range: 62% to 94%). The percentages of agreement for the overall and the individual cases are detailed in Table 2.

The average kappa for the agreement between radiologist 1 and the automated selection was 0.49 (range: 0.04 to 0.87), which corresponds to a moderate agreement, based on the commonly used scale [8] (included for reference in Table 4). The average kappa for the agreement between radiologist 2 and the automated selection was 0.45 (range: −0.13 to 0.81), which also corresponds to a moderate agreement (Table 4). The average kappa between radiologist 1 and radiologist 2 was 0.57 (range: 0.18 to 0.87), which again falls in a moderate agreement category (Table 4). The kappa values and corresponding commonly used agreement categories based on kappa statistics for the individual cases are presented in Tables 3 and 4, respectively.

**Figure 9 fig9:**
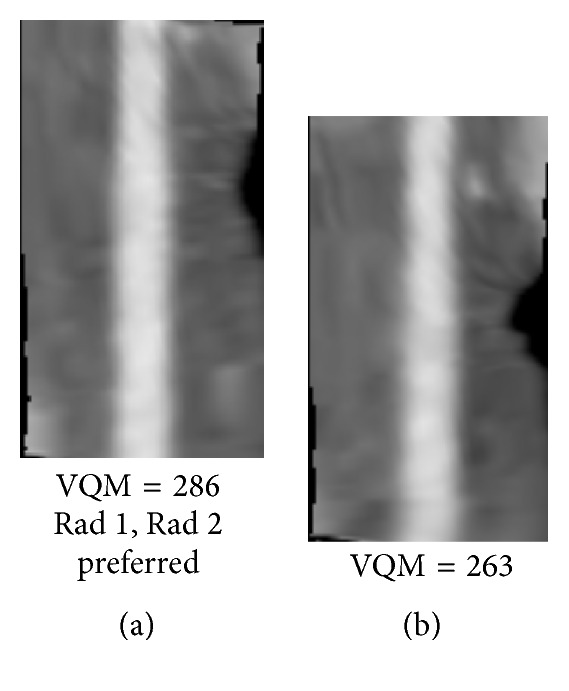
Automatically selected pair of best- and worst-quality vessel branches from the available corresponding arterial branches in six phases using VQM and radiologists' preferences. The automated selection matched the radiologists' preferences (branch (a)).

**Figure 10 fig10:**
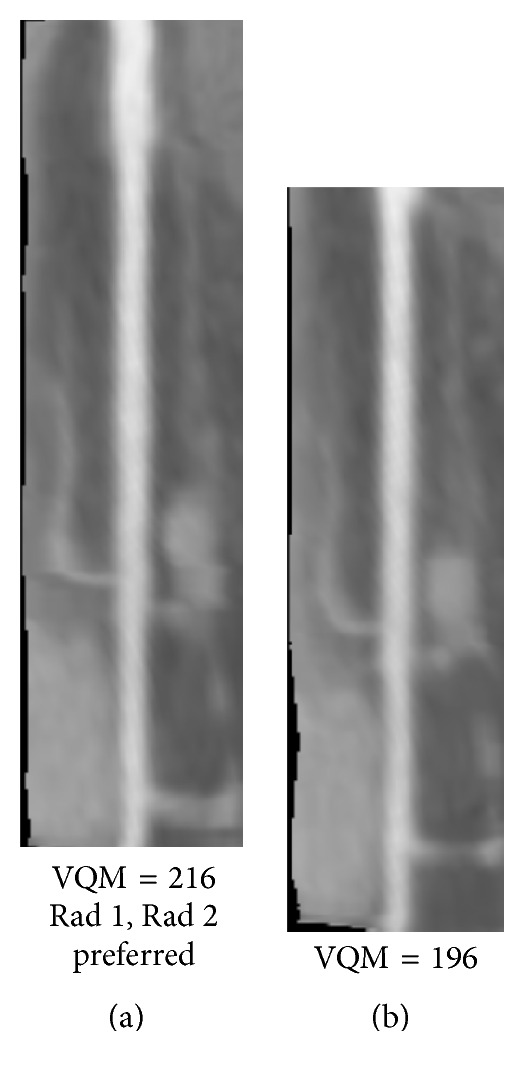
Automatically selected pair of best- and worst-quality vessel branches from the available corresponding arterial branches in six phases using VQM and radiologists' preferences. The automated selection matched the radiologists' preferences (branch (a)).

**Figure 11 fig11:**
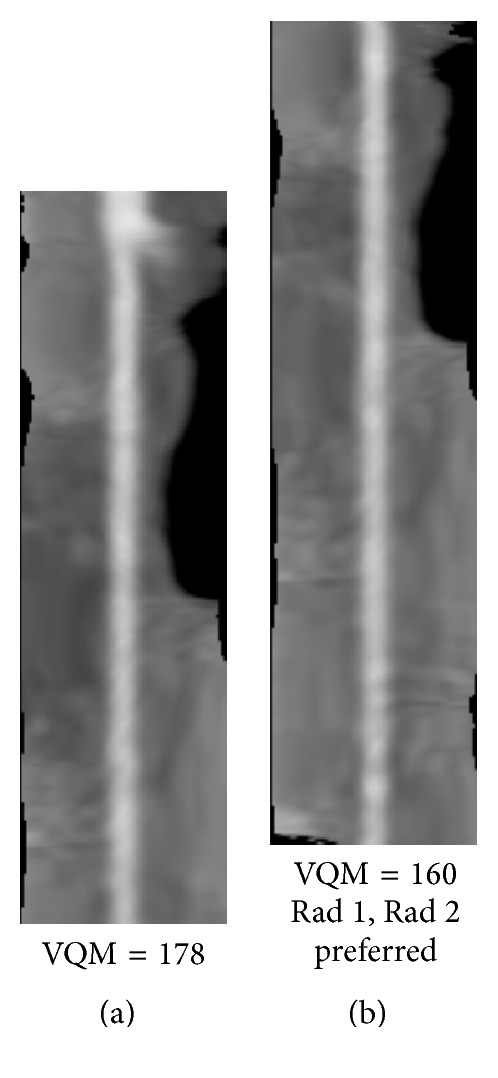
Automatically selected pair of best- and worst-quality vessel branches from the available corresponding arterial branches in six phases using VQM and radiologists' preferences. The automated selection (branch (a)) did not match the radiologists' preferences (branch (b)).

**Figure 12 fig12:**
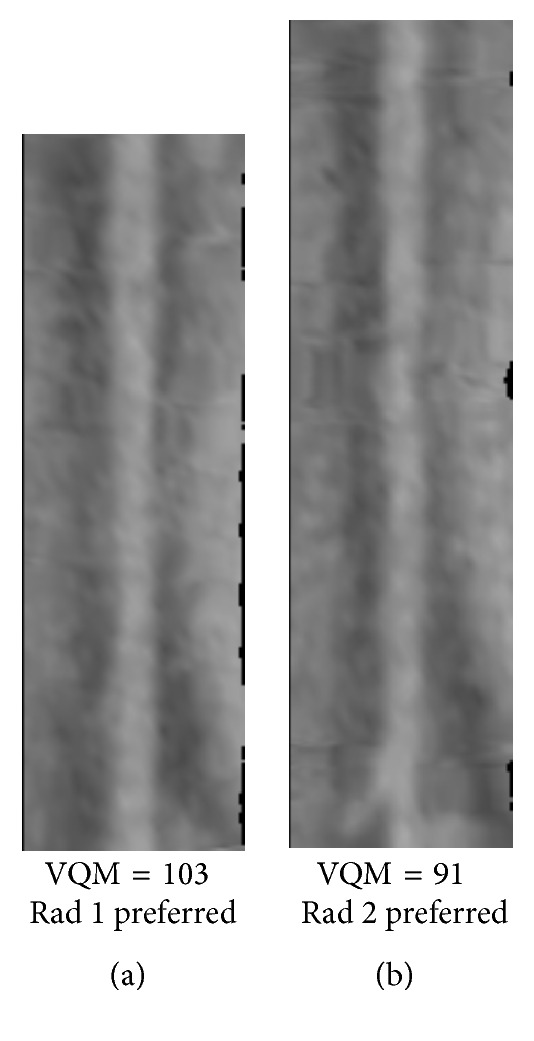
Automatically selected pair of best- and worst-quality vessel branches from the available corresponding arterial branches in six phases using VQM and radiologists' preferences. The automated selection (branch (a)) matched the preference of radiologist 1 but did not match the preference of radiologist 2 (branch (b)).

**Figure 13:**
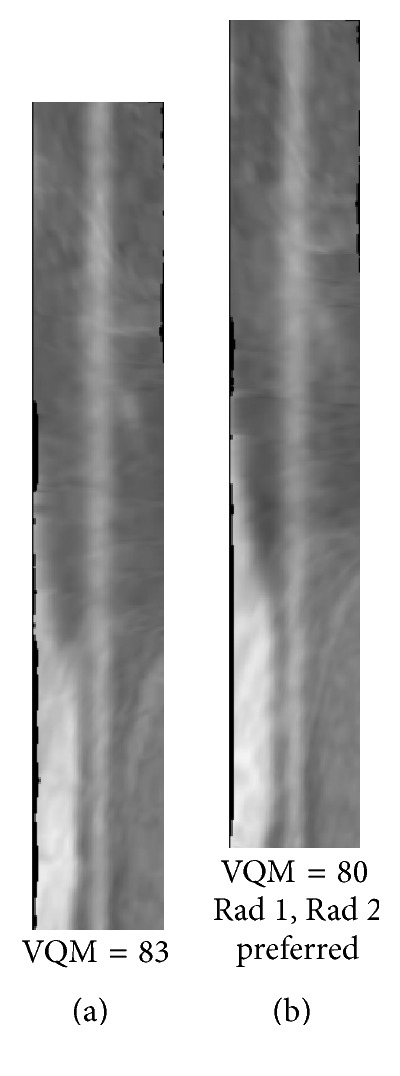
A pair of best- and worst-quality vessel branches selected automatically from the corresponding arterial branches in six phases using VQM and the radiologists' preferences. The automated selection (branch (a)) and the radiologists' preferences (branch (b)) did not match in this case.

Figures 8 [Fig fig9] [Fig fig10] [Fig fig11] [Fig fig12]–13 show examples of the automatically selected pairs of best- and worst-quality vessel branches from available corresponding arterial branches in six phases using VQM and radiologists' preferences. To facilitate comparison, the examples of corresponding branch pairs in Figures 8 [Fig fig9] [Fig fig10] [Fig fig11] [Fig fig12]–13 always show the image with the highest VQM on the left and the image with the lowest VQM on the right. This is different from the randomized left and right placement of the image pairs for the observer study.

## 4. Discussion

In this study, we used cCTA cases with 6 phases for evaluation of the best-quality vessel selection method and the cases were acquired with retrospective gating techniques. Prospective gating, if applicable, is the current state of the art that provides sufficient quality scans with less radiation. However, prospective gating techniques can be effective only in appropriately selected cases when the heart rate is stable with low beat-to-beat variations and is below about 65 bpm. This allows acquisition in a selected phase of the cardiac cycle. If these conditions are not met, retrospective gating is still used clinically to generate cCTA of multiple cardiac phases. In addition, even with prospective gating, more than one phase may be reconstructed, depending on the amount of padding used. Our proposed method can be applied to cCTA examinations with more than one phase and should be independent of whether the multiple phases are obtained by retrospective or prospective gating techniques.

**Figure 14:**
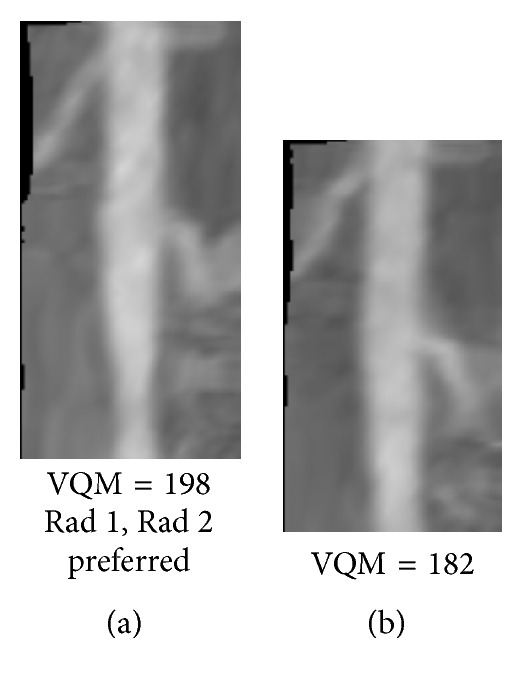
Automatically selected pair of best- and worst-quality vessel branches from the available corresponding arterial branches in six phases using VQM and radiologists' preferences. The automated selection matched the radiologists' preferences (branch (a)).

**Figure 15:**
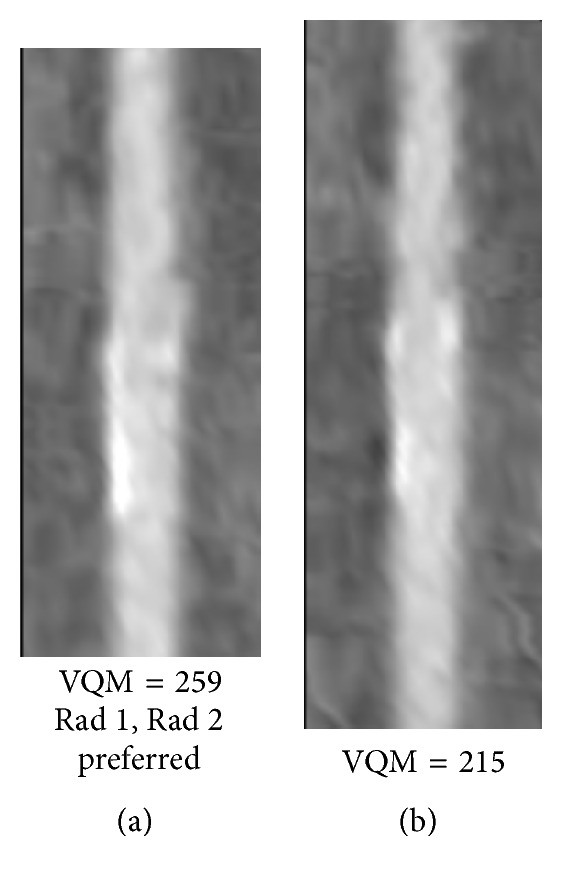
Automatically selected pair of best- and worst-quality vessel branches from the available corresponding arterial branches in six phases using VQM and radiologists' preferences. The automated selection matched the radiologists' preferences (branch (a)).

The image pairs of three branches of different quality are shown in Figures 8 [Fig fig9]–10. The difference in the quality in a pair between the best- and worst-quality vessel branches was relatively similar in these examples. The difference in the VQM of the two branches in the pair in Figure 8 was slightly higher compared to the difference in the pairs in Figures 9 and 10. For all three pairs the automated selection matched the preference of both radiologists. In Figure 11, however, the automated selection (branch (a)) did not match the radiologists' preferences (branch (b)). In Figure 12, the automated selection (branch (a)) matched the preference of radiologist 1 and did not match the preference of radiologist 2 (branch (b)), which was an indication that the difference in the quality of the two branches was very small and the radiologists themselves differed in their quality preference choice. In Figure 13, the automated selection (branch (a)) again did not match the radiologists' preference (branch (b)). Visual inspection shows that the quality of the two branches is very similar, which is also reflected by the very close VQM values (83 and 80, resp.). In this case, as in the case above, the selection of any one branch from the pair would probably be acceptable. Figures 14 and 15 show a vessel pair with branches and a vessel pair with calcified plaques, respectively. The automated selection matched the preferences of both radiologists in both cases. The VQM was able to provide good estimate of the relative vessel quality in these cases, probably because the averaging over the vessel segment made it less sensitive to distortions caused by branches and plaques.

The agreement between the radiologists for Cases 2 and 6 (Table 3) in terms of kappa was lower than the agreement between the automated selection and the radiologists. The percent agreement between the radiologists for Cases 2 and 6 was also lower than or equal to the agreement between the automated selection and the radiologists (Table 2).

The low kappa for the agreement between the radiologists for a case, such as Case 7, indicates that the quality between the best and the worst branch for many vessel pairs in the case is relatively close and it is difficult to make a definitive decision. This is consistent with the near zero and the negative kappa for the agreement between the automated selection and the radiologists. For these vessel pairs, the selection of a branch from any one of the two phases will probably be acceptable, as demonstrated by the examples in Figures 12 and 13.

The average agreements between the automated selection and radiologist 1 and between the automated selection and radiologist 2 were very close in terms of both percent agreement and kappa. The average agreement between the two radiologists was slightly higher than the average agreement between the automated selection and either radiologist.

In this preliminary study, we used only the average radial gradient along the vessel branch as the vessel quality measure. The average radial gradient estimates the sharpness of the vessel wall. It can be expected that other measures such as the contrast and smoothness of the vessels may also be useful as descriptors for the quality of the vessels. We will further develop the VQM to improve the accuracy of the automated ranking of the corresponding branches from multiple phases.

One limitation of the study is the small number of cases. Although this pilot study did demonstrate the feasibility of our approach, a larger data set and more observers have to be used in future studies to further develop and validate the robustness of the methods. A second limitation is that, in clinical practice, cCTA interpretation is not based on one view and all display formats (straightened multiplanar reformation (MPR)s, curved MPRs, and original axial data) are available for diagnosis. However, in our observer study for assessment of the performance of the automatic vessel selection method, the observers were provided with the straightened MPR display to visually judge the vessel quality based on information similar to that used by the computer. This experimental design reduced the reading time to a more practical level for the pilot study and also focused the comparison on the vessel selection stage based on the VQM rather than the accuracy of the entire process including vessel segmentation and straightening. It may also be noted that should the proposed method become practical for clinical use, it would only be used for initial selection of the best phase for a given vessel segment; all the possible (or the preferred) formats of the vessel segment at the selected phase could be displayed automatically so that the radiologist could make use of all diagnostic information as desired. A third limitation is that we did not include all possible branch pairs from all phases in the observer study because the total number of pairings to be evaluated would be over 2000, which would impose excessive demand on the radiologists' effort. Since the comparison of the best- and worst-quality phases was an easier task, the observer study could not reveal the performance of the automatic ranking among all phases. Nevertheless, the observer study did show a correlation of the magnitude of the difference in VQM with the visual similarity in the vessel quality and that small differences in VQM would indicate very similar quality so that the choice of one or the other might not be as critical. We will further improve the methods and perform more extensive validation studies in the future.

## 5. Conclusion

In this study we proposed a method for automatic selection of the best-quality vessels from multiple cCTA phases. The method utilizes a number of image analysis techniques specifically designed for cCTA, including coronary arterial tree segmentation and registration, identification of coronary tree branches and their correspondence among the multiple phases, and assessment of vessel quality by a quantitative measure that guides the selection of the best-quality phase for each vessel from multiple cCTA phases. An observer study with two cardiothoracic radiologists as observers was conducted to evaluate the proposed method. The results demonstrate that the automatic method agreed well with the radiologists' selections and thus the feasibility of the approach. The best-quality arterial segments constitute the building blocks for a virtual best-quality composite coronary arterial tree. The automated selection of the best-quality arterial segments from all available phases of the cCTA is expected to improve the efficiency and facilitate the detection of plaques by either the radiologist or a CAD system.

## Figures and Tables

**Table 1:** Corresponding branch image pairs for the 7 cases.

Case #	Total	LCA	RCA
1	31	17	14
2	14	11	3
3	25	12	13
4	38	23	15
5	18	13	5
6	26	16	10
7	15	10	5

Total	167	102	65

**Table 2:** Agreement between radiologist 1 and the automated selection using VQM of the best-quality branch in the corresponding vessel pairs, between radiologist 2 and the automated selection, and between the two radiologists.

	Number of branches	% agreement		
		Rad 1-computer	Rad 2-computer	Rad 1-Rad 2
Case 1	31	90%	90%	94%
Case 2	14	93%	93%	86%
Case 3	25	84%	76%	84%
Case 4	38	79%	74%	82%
Case 5	18	61%	67%	83%
Case 6	26	62%	77%	62%
Case 7	15	53%	47%	80%

Overall	167	76%	76%	81%

**Table 3:** Cohen's kappa statistics estimation of the agreement between radiologist 1 and the automated selection using VQM of the best-quality branch in the corresponding vessel pairs, between radiologist 2 and the automated selection, and between the two radiologists.

	Number of branches	Kappa		
		Rad 1-computer	Rad 2-computer	Rad 1-Rad 2
Case 1	31	0.87	0.80	0.87
Case 2	14	0.81	0.81	0.65
Case 3	25	0.68	0.51	0.68
Case 4	38	0.56	0.50	0.61
Case 5	18	0.24	0.33	0.67
Case 6	26	0.21	0.32	0.18
Case 7	15	0.04	−0.13	0.33

Overall	167	0.49	0.45	0.57

**Table 4:** Agreement between radiologist 1 and the automated selection using VQM of the best-quality branch in the corresponding vessel pairs, between radiologist 2 and the automated selection, and between the two radiologists estimated in terms of the commonly used categories based on Cohen's kappa statistics [8].

	Kappa		

	Rad 1-computer	Rad 2-computer	Rad 1-Rad 2

Case 1	Almost perfect	Substantial	Almost perfect
Case 2	Almost perfect	Almost perfect	Substantial
Case 3	Substantial	Moderate	Substantial
Case 4	Moderate	Moderate	Substantial
Case 5	Fair	Fair	Substantial
Case 6	Fair	Fair	Slight
Case 7	Slight	Less than chance	Fair

Overall	Moderate	Moderate	Moderate

Agreement categories based on Cohen's kappa statistics [8].

Kappa < 0: less than chance agreement.

Kappa 0.01–0.20: slight agreement.

Kappa 0.21–0.40: fair agreement.

Kappa 0.41–0.60: moderate agreement.

Kappa 0.61–0.80: substantial agreement.

Kappa 0.81–0.99: almost perfect agreement.
